# The Role of Brain-Derived Neurotrophic Factor (BDNF) in the Relation between Physical Activity and Executive Functioning in Children

**DOI:** 10.3390/children9050596

**Published:** 2022-04-22

**Authors:** Julie Latomme, Patrick Calders, Hilde Van Waelvelde, Tineke Mariën, Marieke De Craemer

**Affiliations:** 1 Department of Movement and Sports Sciences, Ghent University, 9000 Ghent, Belgium; 2 Department of Rehabilitation Sciences and Physiotherapy, Ghent University, 9000 Ghent, Belgium; patrick.calders@ugent.be (P.C.); hilde.vanwaelvelde@ugent.be (H.V.W.); tinekemarien@gmail.com (T.M.); marieke.decraemer@ugent.be (M.D.C.)

**Keywords:** physical activity, exercise, brain-derived neurotrophic factor, executive functioning, children

## Abstract

Physical activity (PA) can improve children’s executive functioning (EF), which might be caused by increased levels of brain-derived neurotrophic factor (BDNF). This study investigated whether acute and/or chronic PA leads to increased BDNF levels and enhanced EF in children. **Methods:** In total, 47 children (mean age 9.69 ± 0.60; 46.8% boys) participated. Children performed a maximal exercise test to measure acute PA. Before and after, BDNF was collected and EF was measured. Chronic PA was proxy-reported. Repeated Measures ANOVAs were performed to study the effect of acute PA on BDNF and EF. Mediation analyses were performed to investigate the mediation effect of BDNF on the association between chronic PA and BDNF. **Results:** A borderline significant effect of acute PA on BDNF was found (F = 3.32, *p* = 0.075) with an increase in BDNF (+29.58 pg/mL) after acute PA. A significant effect was found for performance on inhibition tasks (Flanker (accuracy +5.67%, *p* = 0.034) and Go/No-Go (+0.15%, *p* = 0.022)). No effect of acute PA was found on the EF outcomes. No significant correlation between chronic PA and EFs nor BDNF was found. **Conclusions:** Acute PA might increase BDNF and improve some EFs (i.e., inhibition) in children. Chronic PA was not associated with EF nor BDNF. **Trial Registration Number:** NCT02503579.

## 1. Background

The relationship between physical activity (PA) (defined as any bodily movement produced by skeletal muscles that results in energy expenditure) and cognitive functioning (which refers to multiple important mental abilities, including learning, thinking, reasoning, memory, problem solving, decision making, and attention) in children has recently received increased attention [1,2]. More specifically, PA is associated with a variety of physical, physiological, and psychological health benefits [3,4], including a positive impact on children’s cognitive performance [5,6], which is essential for healthy child development [7] and academic performance [5].

Therefore, in several systematic reviews and meta-analyses [7,8,9], PA has emerged as a promising approach to improve children’s cognitive functioning, and more specifically their executive functions (EFs). EFs are a set of higher-order cognitive functions that are responsible for initiating, adapting, regulating, monitoring, and controlling information processes and behavior [10,11]. EFs are furthermore supposed to be the underlying skills for mental and physical health, success in school, and cognitive, social, and physiological development in children [12,13]. Despite this, there is still discussion in the literature on what the specific effects are of PA on children’s cognitive performance [5,14]. This might be due to inconsistencies in the parameters and definitions of PA used across studies, and the mechanisms underlying this association are still insufficiently explored and understood [15].

Regarding the definition of PA, both “acute” PA and “chronic” PA have shown to have beneficial effects on cognitive outcomes [16,17,18], although there is a clear distinction between the two types of PA on cognitive functioning [16]. Whereas acute PA denotes single bouts of PA provoking instant changes in cognitive functioning, chronic PA includes multiple sessions or habitual PA, provoking more long-term cognitive changes [19]. In the literature, however, the difference between the two types of PA is not clear, where the two types are often used interchangeably. Second, it is important that PA is not confused with physical fitness (PF), which is a state of health and well-being and the ability to perform aspects of sports, occupations, and daily activities, and is aimed to be maintained or improved by PA [20]. Given that children’s PA levels are often inconsistent, increases in PA may not necessarily result in enhanced physical (i.e., aerobic) fitness [21]. Therefore, the indirect relationship that exists between children’s PA levels and PF [22] may have contributed to the inconsistent findings in the relationship between children’s PA levels and cognitive outcomes and needs clarification.

Regarding the underlying mechanism, it is hypothesized that the positive effect of PA on cognitive functioning is partly caused by physiological changes in the body, such as increased levels of brain-derived neurotrophic factor (BDNF), that facilitate learning and maintain cognitive functions by improving synaptic plasticity, act as a neuroprotective agent, increase brain circulation, and improve neuro-electric functionality [11,23,24]. However, research on this is very limited in children. Moreover, studies have shown that after acute PA, BDNF levels are increased for a short time period (i.e., explaining increased levels of arousal, attention, and effort), which positively influences the performance on cognitive tasks [17,18]. However, after chronic PA, BDNF levels may potentially remain elevated, and therefore cause a structural effect on neurons, such as neurogenesis and synaptic plasticity. In adults and older adults, it has already been demonstrated that PA alters specific brain functions and changes, which in turn positively influences their cognitive performance [25,26,27]. However, the underlying (brain) mechanism explaining the positive association between chronic PA and cognitive functioning in children is not yet fully understood and studies on this are scarce.

Taken together, the aim of the present study is twofold: First, to investigate whether acute PA leads to increased neurotransmitter secretion (i.e., increased BDNF levels), and enhanced performance on cognitive (executive functioning) tasks, specifically in children. Second, investigate whether chronic PA (not to be confused with PF or acute bouts of PA) leads changes in BDNF levels and, consequently, better executive functioning in children, compared to children that do not engage in chronic or habitual PA. To investigate this assumption, we examined the mediating role of BDNF in the association between chronic PA and executive functioning of children. Regarding the first research question, we hypothesized a better executive functioning on children after acute PA. For the second research question, we expected that in children who are more chronically physically active (i.e., engage in habitual PA) have better executive functioning compared to children who do not engage in chronic PA, due to the chronically increased BDNF levels which lead to structural effects on neurons and synaptic plasticity.

## 2. Methods

### 2.1. Recruitment

In September 2015, healthy subjects were recruited from the primary school WAVO in Onze–Lieve–Vrouw–Waver in Flanders (Belgium). The participants were informed about the aims of the study and received detailed information about the goal of the study, the protocol that would be followed, and the risks and procedures of the study. All parents and children signed an informed consent. The study was approved by the Ethical Committee of the University Hospital Ghent (B670201524725; 29 June 2015).

### 2.2. Procedure

**Figure 1:**
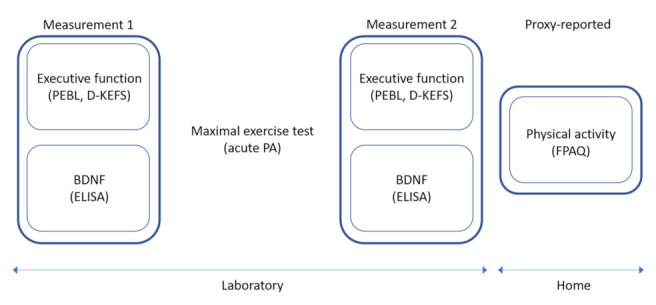
Overview of the different measurements that were conducted (FPAQ = Flemish Physical Activity Questionnaire; BDNF = Brain-Derived Neurotrophic Factor; PEBL = the Psychology Experiment Building Language test battery; D-KEFS = Delis-Kaplan Executive Function System test battery; ELISA = Enzyme-Linked Immunosorbent Assay).

In September 2015, children performed a maximal physical exercise test on an Ergoline 200 cycle ergometer. Before and after the exercise test, children’s BDNF was collected, and their executive functioning was measured with a written and computer task. In addition, children’s physical activity was proxy-reported by the parents using questionnaires. A visual overview of the measurements is displayed in Figure 1 and the different measurements are explained in detail below.

### 2.3. Measures

Before and after the physical exercise test, a blood sample (7 mL) was collected by a study nurse between 9 a.m. and 3 p.m., ±5 min pre- and post-exercise. Blood samples were taken from antecubital veins, placed into plasma venipuncture tubes, separated by centrifugation (3000 rpm for 10 min at 4 degrees Celsius), and stored at 70 degrees Celsius until use.

**Acute physical activity** was measured with a maximal physical exercise test. Based on the weight of every participant, the slope of the cycle ergometer had been set at the weight/4/minute. The respiratory exchange ratio (RER) is the ratio between the amount of carbon dioxide (CO_2_) produced in metabolism and oxygen (O_2_) used. Humans typically inhale more molecules of oxygen than they exhale of carbon dioxide. The ratio is determined by comparing exhaled gasses to room air. The value, however, can exceed 1 during intense exercise, as CO_2_ production by the working muscles becomes greater and more of the inhaled O_2_ gets used rather than being expelled. When the value of 1 was reached, the protocol stopped and the VO_2_ peak and maximal heart rate could be determined. VO_2_max is the point at which oxygen uptake no longer increases (or increases only marginally) with an increase in workload. In the case that a plateau in oxygen uptake is never reached, there is a submaximal exercise test in which VO_2_peak is recorded.

**Chronic physical activity** was proxy-reported by the parents using the valid and reliable Flemish Physical Activity Questionnaire (FPAQ) [28]. Total physical activity (expressed in minutes/week) was calculated by making the sum of five variables (V): V1 (minutes/week of physical education at school) + V2 (minutes/week of other physical activities and sports performed at school) + V3 (minutes/week of physical activities and sports performed in leisure time) + V4 (minutes/week of active transport to school) + V5 (minutes/week of active transport in leisure time).

**BDNF** before and after the physical exercise test was derived from the serum using Quantikine Enzyme-Linked Immunosorbent Assay (ELISA) Human BDNF Immunoassay kits (Bio-Techne). It is a 3.5 h solid phase ELISA designed to measure free, human BDNF in serum. It contains recombinant human BDNF expressed in S*f*21 cells and antibodies raised against the recombinant factor. This immunoassay quantitated the recombinant BDNF accurately (Human Free BDNF Quantikine ELISA (Available online: bio-techne.com (accessed on 17 August 2021))). The quantitative sandwich enzyme immunoassay technique was used. A monoclonal antibody specific for human free BDNF was pre-coated onto the microplate. Standards and samples were pipetted into the wells, and free BDNF that is present is bound by the immobilized antibody. An enzyme-linked monoclonal antibody specific for human, free BDNF was added to the wells. Then, a wash is carried out to remove any unbound antibody-enzyme reagent, after which a substrate solution is added to the wells and color develops in proportion to the amount of free BDNF bound in the initial step. The color development was then stopped, and the intensity of the color was measured. A 96-well polystyrene microplate (12 strips of 8 wells) was used, and the resulting absorbance was read in duplicate using a microplate reader at 450 nm. All further details regarding the measurement of BDNF can be found online (Human, Free BDNF Quantikine ELISA (Available online: bio-techne.com (accessed on 17 August 2021))). Eventually, BDNF was expressed as (picogram (pg) per milliliter (mL)).

**Executive functioning** was measured using written and computer tasks, using, respectively, the Delis–Kaplan Executive Function System [29] (D–KEFS) and the Psychology Experiment Building Language (PEBL) computerized test battery (Available online: http://pebl.sourceforge.net/ (accessed on 17 August 2021)). The written tasks of the D–KEFS were the Trail-Making Test, the Design Fluency Test, and the Stroop Color–Word Test. The PEBL computer tasks were: The Go/No-Go test, the Flanker test, and the Corsi Block-Tapping Test. Each test will now be explained in more detail.

- *The Flanker Test*: this test measures inhibition by measuring the effect of conflicting information within a stimulus set. The child receives an image of five arrows and should only look at the arrow in the middle. If the middle arrow is pointing right, then the right shift key should be pressed. If the middle arrow is pointing left, then the left shift key should be pressed. The other arrows are causing conflicting information. The outcomes of the Flanker test are mean accuracy and mean reaction time.
- *The Go/No-Go Test:* The child sees four different squares on the screen. In one of the four squares, either the letter ‘P’ appears or the letter ‘R’. When the letter P appears, the child should press the right shift button. When the letter ‘R’ appears, the child should not press a key or click on the mouse. The response accuracy of each No-Go trial is used as a measurement for inhibitory control. The outcomes of the Go/No-Go test are expressed as mean accuracy and conflict–cost reaction time.
- *The Corsi Block-Tapping Test:* The Corsi Block-Tapping Task is a widely used test to assess visuospatial working memory. The child sees nine blocks on the screen. The blocks light up one by one in a random order. The child needs to replicate the order of the blocks. The first trial lights up two blocks, the next trial lights up three blocks, and each trial gradually increases until there are nine blocks in length. The outcome of the Corsi Test was the total score which reflects the total number of correct blocks replicated.
- *Trail-Making Test.* The D–KEFS Trail Making subtest (Delis et al., 2001) contains five conditions. The first and fourth conditions were a focus of this study. During the first condition, “Visual Scanning”, the participant is asked to cross-out circles that contain a particular number. On the fourth condition, “Number–Letter Switching”, the participant is asked to draw a line connecting dots, alternating between dots containing numbers and dots containing letters. This condition is a commonly used measure of set-switching, or cognitive flexibility, and has high reliability (Delis et al., 2001). For this study, the scaled scores of completion time in each condition were used as a measure of visual scanning and memory (condition 1) and switching/cognitive flexibility (condition 4), with lower scores indicating better performance.
- *Color*–*Word Interference Test.* This D–KEFS subtest consists of four parts: color naming (condition 1), word reading (condition 2), inhibition (condition 3), and inhibition/switching (condition 4). The inhibition trial is the third condition, which was the focus of this study. In this condition, the participant is presented with a page containing the words “red,” “green,” and “blue” printed incongruently in red, green, or blue ink. The participant is asked to say the color of the ink in which each word is printed as quickly as he/she can without making mistakes. Performance is measured by accuracy and reaction time on this condition.
- *Design Fluency Test.* The D–KEFS Design Fluency (DF) Test consists of three trials in which participants create novel designs by connecting dots in a series of five dot matrices. The three conditions are referred to as Filled Dots (connecting filled dots), Empty Dots (connecting empty dots while filled dots function as distractors), and Switch (switching between connecting filled and empty dots). The third condition includes switching, which was examined in the current study, resulting in a score representing the total number of correct unique designs/patterns generated during the “switch” condition.

## 3. Data Analysis

Data of 47 children were included for this study. All participants were included since they had complete data on both outcome variables (i.e., BDNF and at least one executive functioning variable). Descriptive statistics were computed to describe the sample characteristics, using IBM SPSS Statistics for Windows, version 26.0 (New York, NY, USA) [30].

**Figure 2:**
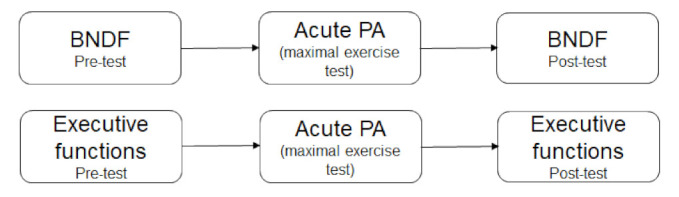
The effect of acute PA on (1) BDNF levels and (2) executive functions.

For investigating the effect of acute PA on BDNF levels and EFs (see Figure 2), Repeated Measures ANOVAs with Time as the within-factor variable and BDNF and executive functions as the dependent (outcome) variables were conducted. Age, sex, and BMI-z scores were added as confounders in the statistical models to control for their confounding effect.

**Figure 3:**
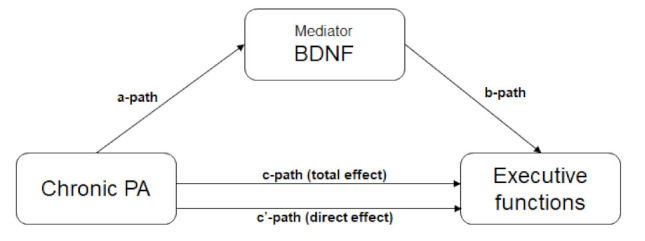
The mediation effect of chronic PA on EFs through BDNF levels.

For investigating the mediation effect of chronic PA on EFs, we first checked whether the assumptions were fulfilled to establish a mediation effect, as recommended by Baron and Kenny, i.e., whether: (i) predictor and outcome are significantly associated, and (ii) in order to include them in the model, mediators are significantly associated with both predictor and outcome variables. IBM SPSS Statistics for Windows (v26.0; New York, NY, USA) was used to conduct Pearson correlation analyses between the mediator (i.e., BDNF), the predictor (chronic PA), and the outcome variables (EF variables) to check the aforementioned assumptions. Using model 4 of an SPSS macro provided by Preacher and Hayes (2008) (42), mediation analyses with one mediator (i.e., BDNF) was conducted to evaluate whether the association between chronic PA and the different executive functions were mediated by BDNF. When the effect of chronic PA on a certain executive function of the child (direct effect) is removed (i.e., complete mediation) or reduced (i.e., partial mediation) when controlled for the mediator, then a mediation effect occurs. However, before mediation analysis can be performed, a significant association between predictor and outcome variables is required. In addition, the mediator should be significantly correlated with both the outcome variable and the predictor. If all conditions are met, four effects can be estimated in the mediation model (see Figure 3): (1) the total effect (c-path), showing the effect of chronic PA on a certain executive function; (2) the direct effect (c’-path), showing the direct effect of chronic PA on a certain executive function; (3) two indirect effects, i.e., the effect of chronic PA on the mediator BDNF (a-path) and the effect of the mediator BDNF on a certain executive function (b-path); and (4) the mediation effect, which is the effect of chronic PA on a certain cognitive function, via the mediator BDNF.

## 4. Results

### 4.1. Descriptive Statistics

In total, 47 children were included in the final dataset. Mean age of the sample was 9.69 years old (SD = 0.60), and 46.8% (*n* = 22) were boys. Mean total PA per week was 465.70 (SD = 231.71, range: 150–1030) minutes per week, which corresponds to 66 min/day. Mean BMI z-score was −0.22 (=normal weight) (SD = 1.01, range: −1.88–2.31).

### 4.2. Research Question 1. Effect of Acute PA on BDNF and EFs

The Repeated Measures ANOVA showed a tendency to a significant effect of acute PA on BDNF levels (F = 3.32, *p* = 0.075). More specifically, BDNF levels increased with 29.58 pg/mL after acute PA. For EFs, the Repeated Measures ANOVAs showed a significant effect of acute PA on some EFs. More specifically, performance (i.e., accuracy) on the PEBL Flanker Test and the PEBL Go/No-Go Test increased with 5.67% and 0.15%, respectively. after acute PA. No significant effects were found for the other PEBL tasks (i.e., Digit Span Test and Corsi Test), nor on the D–KEFS tasks (i.e., the Color–Word Interference Test, Design Fluency Test, and Trail-Making Test) (Table 1).

### 4.3. Research Question 2. Chronic PA and (Mediation) Effect on BDNF and EFs

#### Correlation Analysis

The correlation analysis showed no significant association between the predictor (chronic PA) and any of the outcome variables on the pretest measurements (i.e., EFs before the maximal exercise test), neither between the mediator (BDNF) nor any of the outcome variables required for conducting a mediation analysis. Therefore, no mediational effect could be tested. The results of the correlation analysis can be found in Table 2.

## 5. Discussion

The aim of the current study was twofold. Firstly, we looked at the association between acute PA and EFs in children (8–10 years) and acute PA and BDNF levels, where we expected increased BDNF levels and, consequently, executive functioning. Secondly, we examined whether chronic PA (i.e., habitual PA or high levels of total PA on a weekly basis) was associated with EF and whether this association was mediated by increased BDNF levels.

The results of the first research question showed a borderline significant increase in BDNF, and a significant and positive change in the Flanker Test and the Go/No-Go Test. Although the increase in BDNF was only borderline significant, it is expected that increasing the sample size might increase significance. BDNF increased with ± 20.00 pg/mL, which is already clinically important and similar to increases found after aerobic exercise in previous studies [31]. In the current study, BDNF was derived from serum, which means that peripheral concentrations of BDNF were assessed. Although BDNF can also be derived from other tissues, such as immune, liver, smooth muscle, and vascular endothelial cells, BDNF measured in peripheral tissues is a good indicator of BDNF activity in the brain. It is important to mention that there is a nonsynonymous single nucleotide polymorphism in the gene encoding BDNF val66, which has been related to cognitive function and brain morphology [32]. In this polymorphism, a methionine-specifying allele, as compared with the alternate valine-specifying allele, is associated with decreased levels of BDNF-protein secretion and distribution [33]. However, this polymorphism could not be detected in the lab, which might be a reason for our borderline significant result since BDNF is closely linked to PA [34].

The accuracy measured with the Flanker Test improved with 5.67%, which could be interpreted as clinically relevant. However, the accuracy measured with the Go/No-Go Test improved with 0.15%, which was statistically significant, but too small to be considered to be clinically important. Both tests assess inhibition, reflecting the capacity to obstruct automatic or dominant responses when they are not appropriate for the context at hand [10,35,36] and the ability to suppress the influence of interfering information [37,38]. The handling of task-irrelevant information is of great importance for memory processes, information processing skills, and the interplay of information selection. The findings of the present study are in line with previously conducted research, where studies that employ tasks measuring response inhibition provide compelling evidence for the influence of acute bouts of exercise on the ability to block irrelevant information and to select and respond to task-relevant information [39,40]. Furthermore, Diamond et al. (2007) have revealed that stimulating EFs within the school context, and more specifically inhibition, leads children to greater academic success [41]. In this line, studies have shown that measures of inhibition are predictive for early academic skills, such as written expression, reading skills, and mathematical tasks [42,43].

During the acute maximal exercise test between both measurements of EF and BDNF, no cognitive elements were added. Children had to perform the maximal exercise test, but no cognitive loading was added. However, adding cognitive activity might have an influence on EF. Some intervention studies already investigated the combination of chronic PA and cognitive activity (i.e., brain activity associated with the use of cognitive functions) such as dual-tasking exercises or exergames. A study in preschool children, for example, showed that a combination of PA and cognitive tasks caused an increase in their working memory (updating) performance [44]. The randomized controlled trial study of Schmidt et al. (2020) in 181 primary school children between 10 and 12 years old showed that including cognitive tasks during the performance of PA is promising in increasing EF [44]. A possible strategy in future studies might be to include cognitive tasks during acute PA to study whether there is an increase in BDNF and whether this increase is a mediating factor between acute PA and EF.

The results from our study are in line with previous studies looking into the effect of acute PA on BDNF and cognitive tasks. The studies of Kashihara et al. (2009) and Tomporowski et al. (2003), for example, showed an increase in BDNF levels after acute PA which had a positive effect cognitive performance [17,18]. Up until now, this was only investigated in adults, and thus, our results show that the current findings are also applicable in younger age groups.

Secondly, we aimed to investigate the mediational effect of BDNF on the association between chronic PA on executive functioning. However, we were not able to conduct a mediation analysis since the prerequisites to perform a mediation analysis were not met, as there was no significant correlation between chronic PA and EFs, and also no significant correlation was found between chronic PA and BDNF, and BDNF and EFs. Therefore, we were not able to determine whether BDNF is a mediator of the association between chronic PA and EFs. The absence of an association between chronic PA and executive functioning is in contrast with the literature, in which associations were found between daily PA (total volume of PA and moderate-to-vigorous PA) and executive functioning in primary-school-aged children. One of the reasons for the absence of these associations might be the fact that chronic PA was measured using a proxy-reported questionnaire (i.e., the FPAQ). This questionnaire is a valid and reliable tool to measure children’s PA level, but it is possible that socially desirable answers were given by the parents. This questionnaire was completed by parents and assessed sports and physical education at school, sports during leisure time, and active transportation to school and in leisure time. Since parents were not present during school hours to observe their child, they had to estimate the amount of time spent on these activities which might have induced bias. In addition, the activities mentioned in the questionnaire mainly assess moderate to vigorous PA, which means that the amount of light PA might be missed. Therefore, future studies could look at using objective measurement devices such as accelerometers (e.g., ActiGraph or Axivity) to have an objective measure of total PA, and also an objective measure for the different intensities of PA (i.e., light, moderate, and vigorous) as it might be possible that the intensity of chronic PA also plays an important role. For example, the study of Van der Niet et al. (2015) in 80 8- to 12-year old children, in which physical activity was measured by accelerometry, found differences in the associations between total the volume of PA and EF on the one hand and between moderate-to-vigorous PA and EF on the other hand [45]. Using accelerometers or other types of objective measurement devices opens up the opportunity to investigate the duration and intensity of chronic PA and the association with EF in primary school children.

Besides the strengths, there are also some study limitations. The largest limitation is the lack of a control group. Therefore, it is unclear whether the changes in EFs and BDNF are derived from the acute effect of PA. Future studies should incorporate a control group. Learning effect could not be diminished since the EFs were repeatedly measured within a short period of time. In addition, PA was measured using proxy-reported questionnaires with no distinctions made between the different intensities of PA (i.e., light, moderate, and vigorous PA), and the nonsynonymous single nucleotide polymorphism could not be detected in the lab. Furthermore, it could also be interesting to investigate the association between sedentary behavior and executive functioning in children. The strengths are the fact that a wide range of EF tests were conducted, which made it possible to distinguish between different EFs (i.e., inhibition, attention, visuospatial working memory, switching, and cognitive flexibility). In addition, both chronic and acute PA were measured within this study, which made it possible to study the influence of both chronic and acute PA on EFs.

## 6. Conclusions

This study investigated whether both acute and chronic (i.e., habitual) PA leads to increased neurotransmitter secretion (i.e., increased BDNF levels) and enhanced performance on cognitive (executive) functioning in children, and whether BDNF mediates the association between chronic PA and executive functioning of children. The results showed that acute PA increases BDNF levels and improve some EFs, and more specifically inhibition, in children. These results are in line with previous studies showing an increase in BDNF levels after acute PA, which had a positive effect on cognitive performance. However, up until now this was only investigated in adults; thus, our results show that the current findings are also applicable in children. Chronic PA was, however, not associated with EF nor with BDNF. However, as chronic PA was proxy-reported, which might have induced bias, objective measurements of chronic PA are needed to confirm our findings.

## Figures and Tables

**Table 1:** Results of the effect of acute PA on BDNF and the different executive functions in children.

Executive Functions			Pre-Test (Acute PA) Mean (SD)	Post-Test (Acute PA) Mean (SD)	*p*-Value
Executive Functioning Test	Executive Functioning Component	Measurement			
PEBL					
Flanker Test	Inhibition	Reaction time, in msec	557.00 (106.94)	535.11 (97.70)	*p* = 0.509
		Accuracy, in %	70.99 (15.69)	76.66 (12.96)	*p* = 0.034 *
Go/No-Go Test	Inhibition	Accuracy, in %	84.10 (5.77)	84.25 (7.12)	*p* = 0.022 *
		Reaction time (conflict–cost), in msec	55.16 (61.66)	52.31 (54.27)	*p* = 0.484
Corsi Test	Visuospatial working memory	Total score, *n*	29.96 (10.56)	34.24 (12.78)	*p* = 0.747
**D–KEFS**					
Color–Word Inference Test	Condition 3: inhibition	Reaction time, in msec	82.67 (21.07)	71.61 (24.61)	*p* = 0.624
		Accuracy, in %	93.11 (4.49)	95.50 (4.18)	*p* = 0.795
Design Fluency Test	Condition 3: switching	Correct patterns, *n*	11.23 (2.60)	13.70 (15.39)	*p* = 0.508
Trail-Making Test	Condition 1: visual scanning and memory Condition 4: switching & cognitive flexibility	Completion time, in sec	11.82 (2.46) 11.18 (3.29)	13.16 (2.03) 12.45 (2.93)	*p* = 0.323 *p* = 0.479
**BDNF (in pq/mL)**			28.58 (63.06)	30.59 (88.20)	*p* = 0.075

* *p* < 0.05. All analyses were adjusted for age, gender, and BMI-z scores at baseline.

**Table 2:** Bivariate correlations among chronic PA, executive functions, and BDNF.

	Pre-Exercise Executive Functions									
	PEBL					D-KEFS				
	**Flanker Test (Reaction Time)**	**Flanker Test (Accuracy)**	**Corsi Test (Total Score)**	**Go/No-Go Test (Reaction time)**	**Go/No-Go Test (Accuracy)**	**Color–** **Word Interference Test (Reaction Time)**	**Color–** **Word Interference Test (Accuracy)**	**Design Fluency Test**	**Trail-Making Test** **(C1)**	**Trail-Making Test** **(C4)**
**Chronic PA**	−0.157	0.171	0.046	0.102	0.006	−0.285	−0.141	−0.136	0.091	0.290
**BDNF**	−0.094	0.095	0.064	0.083	−0.039	0.005	0.053	0.191	0.018	0.211

## Data Availability

Data sharing is applicable upon request.
